# Rib kinematics during lung ventilation in the American alligator (*Alligator mississippiensis*): an XROMM analysis

**DOI:** 10.1242/jeb.156166

**Published:** 2017-09-01

**Authors:** Robert J. Brocklehurst, Sabine Moritz, Jonathan Codd, William I. Sellers, Elizabeth L. Brainerd

**Affiliations:** 1 School of Earth and Environmental Sciences, University of Manchester, Manchester M13 9PT, UK; 2 Department of Ecology and Evolutionary Biology, Brown University, Providence, RI 02912, USA; 3 Faculty of Biology, Medicine and Health, University of Manchester, Manchester M13 9PT, UK

**Keywords:** Archosauria, Crocodylia, Breathing, Biomechanics, X-ray, Costovertebral joint, Vertebral rib

## Abstract

The current hypothesis regarding the mechanics of breathing in crocodylians is that the double-headed ribs, with both a capitulum and tuberculum, rotate about a constrained axis passing through the two articulations; moreover, this axis shifts in the caudal thoracic ribs, as the vertebral parapophysis moves from the centrum to the transverse process. Additionally, the ventral ribcage in crocodylians is thought to possess additional degrees of freedom through mobile intermediate ribs. In this study, X-ray reconstruction of moving morphology (XROMM) was used to quantify rib rotation during breathing in American alligators. Whilst costovertebral joint anatomy predicted overall patterns of motion across the ribcage (decreased bucket handle motion and increased calliper motion), there were significant deviations: anatomical axes overestimated pump handle motion and, generally, ribs *in vivo* rotate about all three body axes more equally than predicted. The intermediate ribs are mobile, with a high degree of rotation measured about the dorsal intracostal joints, especially in the more caudal ribs. Motion of the sternal ribs became increasingly complex caudally, owing to a combination of the movements of the vertebral and intermediate segments. As the crocodylian ribcage is sometimes used as a model for the ancestral archosaur, these results have important implications for how rib motion is reconstructed in fossil taxa, and illustrate the difficulties in reconstructing rib movement based on osteology alone.

## Abstract (precis)

**Summary:** Using XROMM to test how well joint anatomy predicts rib motion during breathing in crocodylians, our best living model for the earliest archosaurs.

## INTRODUCTION

The primitive mode of ventilation in amniotes is costal aspiration, which uses the motion of the ribs and sternum driven by the axial musculature to ventilate the lungs (Janis and Keller, 2001; Brainerd, 2015). Crocodylians (extant members of the wider Crocodilia) also possess a suite of derived secondary ventilation mechanisms including: ventral rotation of the pubis; displacement of the gastralia; flexion of the vertebral column; and the ‘hepatic piston’, retraction of the liver and other abdominal viscera through contraction of the diaphragmaticus muscle (Gans and Clark, 1976; Farmer and Carrier, 2000; Claessens, 2004b, 2009b). Of these, the two most important are costal aspiration and the hepatic piston (Movie 1) (Claessens, 2009b).

The mechanics of the hepatic piston are relatively straightforward and have been extensively studied (Farmer and Carrier, 2000; Uriona and Farmer, 2006; Munns et al., 2012), but costal aspiration is more complex. Ventilation is powered by the hypaxial muscles, with the transverse abdominal responsible for exhalation, and the intercostal muscles powering inhalation and exhalation (Gans and Clark, 1976; Carrier, 1989; Brainerd and Owerkowicz, 2006). However, muscles can only do positive work and hence contribute to ventilation when actively shortening, but in order to power inhalation this muscular contraction must be converted into expansion of the thorax (Brainerd and Owerkowicz, 2006; Brainerd, 2015).

Crocodylians, along with almost all other amniotes (turtles are one notable exception; Lyson et al., 2014), possess a mobile ribcage, which forms a musculoskeletal lever system, and it is this lever system that allows the conversion of intercostal muscle shortening into thoracic expansion (Brainerd and Owerkowicz, 2006; Brainerd, 2015). Given their essential role in costal aspiration, it is important to understand the form–function relationship between ribcage morphology and the mechanics of thoracic volume change, particularly in crocodylians, as they serve as our best extant model for fossil archosaurs (Claessens, 2015).

The crocodylian trunk can be divided into a rib-bearing thoracic region and a rib-free lumbar region (Hoffstetter and Gasc, 1969). Each thoracic rib is tripartite, consisting of an ossified vertebral segment and cartilaginous intermediate and sternal segments, which articulate with a cartilaginous sternum (Hoffstetter and Gasc, 1969). Therefore, each rib has four joints: the costovertebral joint between the ribs and vertebrae, the dorsal intracostal joint between the vertebral and intermediate ribs, the ventral intracostal joint between the intermediate and sternal ribs and, finally, the sternocostal joint between the sternal ribs and sternum. Whereas the costovertebral and both intracostal joints are synovial, the sternocostal joints are cartilaginous (Frey, 1988; Claessens, 2009b).

**Fig. 1.**
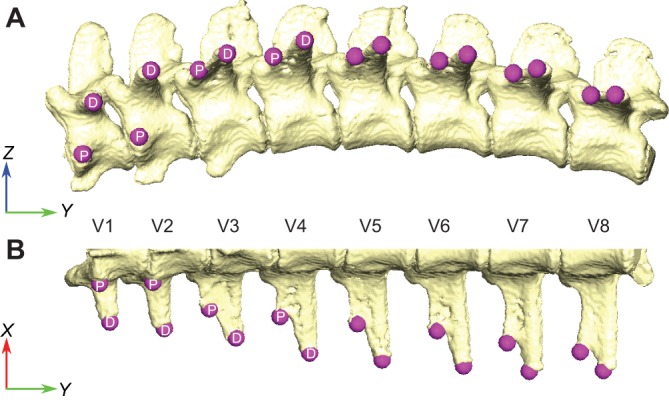
**Vertebral column of *Alligator mississippiensis*.** (A) Lateral and (B) ventral views of a mesh model from a CT scan showing the position of the parapophysis (P) and diapophysis (D). The diapophysis is located on the centrum of the first two vertebrae (V1 and V2) but shifts to the transverse process on V3 and subsequent thoracic vertebrae. The orientations of *X*-, *Y*- and *Z*-axes used to define predicted motions from morphology are shown.

The vertebral ribs are bicapitate, with a distinct capitulum and tuberculum that articulate with the vertebral parapophysis and diapophysis, respectively. The first two thoracic vertebrae have the parapophysis on the vertebral centrum, but in the third thoracic vertebra the parapophysis shifts onto the transverse process, and migrates laterally towards the diapophysis in successively caudal vertebrae (Fig. 1) (Claessens, 2009b; Schachner et al., 2009). This change in the position of the costovertebral articulations is reflected in changes to the rib-head morphology; the first two thoracic ribs have a very distinct dorsal projection for the tuberculum, and the rib heads are strongly forked (Schachner et al., 2009). However, following the migration of the parapophysis onto the transverse process, the tuberculum's dorsal projection is reduced and it more closely resembles a shoulder on the capitulum (Schachner et al., 2009).

List of abbreviations

CT:   computed tomography
JCS:   joint coordinate system
XROMM:   X-ray reconstruction of moving morphology
*ZYX*:   rotation order for JCS Euler angles

Theoretically, the bi-condylar costovertebral joint should behave like a hinge, with the parapophysis and diapophysis forming an anatomical axis of rotation that constrains the motion of the vertebral ribs in crocodylians (Claessens, 2009b); however, this has never been tested experimentally, and any laxity of the joint capsule could allow a combination of sliding movements that would potentially permit additional degrees of freedom in the motion of the rib. It is also not entirely clear how the additional degrees of freedom associated with a tripartite ribcage affect patterns of rib motion, as rotations at the intracostal joints have been only briefly described (Claessens, 2009b). Preliminary X-ray reconstruction of moving morphology (XROMM) analysis of alligator rib mobility found little motion in the dorsal intracostal joint (Moritz and Brainerd, 2013), and so these anatomically tripartite ribs may be functionally bipartite, with the intermediate and vertebral ribs functioning as a single unit. Being cartilaginous, it is also possible that the sternal and intermediate ribs bend during ventilation, similar to the gastralia (Carrier and Farmer, 2000; Claessens, 2009b), and this could provide another mechanism affecting volume change during ventilation.

Previous studies of ventilation in crocodylians used two-dimensional single-plane fluoroscopy (Claessens, 2004b, 2009b); however, a limitation of this method is that it cannot capture details of non-planar motion, and during ventilation the ribs move in a complex, three-dimensional (3D) manner (Brainerd, 2015). XROMM is a relatively new technology that allows truly three-dimensional motion analysis, capable of measuring all possible translations and rotations that may occur across joints during biological motion (Brainerd et al., 2010; Gatesy et al., 2010). XROMM combines skeletal kinematics data from *in vivo* bi-planar X-ray cineradiography, with accurate 3D bone models obtained from CT scans, and allows full 3D motion analysis to be carried out in the context of detailed musculoskeletal morphology.

In the present study, the skeletal kinematics of ventilation in the American alligator [*Alligator mississippiensis* (Daudin 1802)] are analysed using marker-based XROMM. These observations will allow us to investigate whether the axis of rotation of the vertebral ribs in crocodylians *in vivo* is the same as that predicted based on joint anatomy, as well as whether the migration of the parapophysis and the change in the orientation of the costovertebral joint are reflected in differences in the rotations of the vertebral ribs as we look at the cranio-caudal sequence of rib movements. It will also allow us to see whether rotations at the dorsal intracostal joint occur at all, and how these compare with the motion across the other costal joints. Additionally, this will serve as a valuable exploratory study in terms of simply describing how the rib segments move relative to one another, the sternum and the vertebrae compared with other sauropsids (e.g. *Iguana*, *Salvator*), which will also help inform reconstructions of ventilation kinematics in fossil taxa.

## MATERIALS AND METHODS

Data were collected from four 3-year-old (sub-adult) American alligators. The animals were of unknown gender, and body masses ranged from 2.9 to 3.3 kg. All animal care and experimental procedures were approved by the Institutional Animal Care and Use Committee of Brown University. All raw data collected for this study (X-ray video and CT scan data) are available from the X-ray Motion Analysis Portal (xmaportal.org).

The alligators were fasted for at least 24 h prior to the marker implantation surgery. Prior to induction, animals were given an opioid analgesic and then anaesthesia was induced using 3.5% isoflurane in oxygen via a mask. Further analgesia was provided during and after surgery. When ready, the animals were intubated using an appropriately sized cuffed tracheal tube, and anaesthesia was maintained under 2–2.5% isoflurane in O_2_. To allow recovery of normal breathing function post-operatively, animals were allowed to recover for a minimum of 4 days prior to beginning breathing trials.

Radio-opaque markers were implanted to track the motions of the vertebrae, ribs and sternum. Beads on posts [1 mm tantalum beads (Baltec, Los Angeles, CA, USA) with holes laser-drilled into them and mounted on short (2–3 mm) segments of insect pins (0.25 mm diameter)] were used to mark the ribs (Brainerd et al., 2016). To mark the sternum, 1 mm tantalum beads were implanted directly (without posts), and 2 mm tantalum beads were implanted into the scutes along the midline to act as vertebral markers. For beads on posts and solid beads, small holes were drilled into the bone using a hand drill with a diameter matching the marker type, and markers were press-fit into the holes. As only a few ribs could be marked per animal, the vertebral column and sternum were marked in all individuals, and different specimens had different ribs marked to ensure the entire ribcage was covered. For exactly which bones were marked in which individuals, see Table S1.

X-ray videos of ventilation were collected with custom biplanar fluoroscopy equipment housed in the W. M. Keck Foundation XROMM Facility at Brown University (Miranda et al., 2011). Videos were recorded at rates of 60 or 100 frames s^−1^, with an exposure time of 700 μs and X-ray energy set at 70–90 kV and 100–200 mA. A fast shutter speed (low exposure time) was used to darken the images and permit increased X-ray energy and lower noise from the fluoroscopes. Animals were imaged during both quiet and deep breathing, with deep-breathing trials coming after treadmill locomotion or animal handling; animals were not stimulated to breathe deeply by exposure to hypercapnia. Following video data collection, the animals were euthanized by an overdose of pentobarbital (100 mg kg^−1^ delivered intravenously) and computed tomography (CT) scans were collected at Rhode Island Hospital (Philips Medical System, Best, The Netherlands, 80 kV, variable mA, 0.625 mm slice thickness).

### 3D models and anatomical joint axes

From the CT data, 3D mesh models of individual marked bones and the metal markers were created in Avizo (Version 8.1, 9.0, FEI Visualization Sciences Group, Hillsboro, OR, USA). Initial segmentation of bone and markers was done automatically based on differences in greyscale thresholds, and individual bones were then separated from one another manually. The high radio density and small size of the markers created burst-point artefacts in the CT scans, which were manually removed. Polygonal surface mesh models were generated for each bone, as well as a single surface mesh for all the markers, to show their positions relative to the bones. Basic smoothing was applied to all bone and marker models, before they were exported from Avizo as OBJ files. Further smoothing, mesh cleaning and mesh reduction (to reduce file sizes whilst still retaining anatomical accuracy) were carried out in Studio (2012, Geomagic Inc., 3D Systems, Research Triangle Park, NC, USA) and Meshlab (http://meshlab.sourceforge.net/). Finally, models were imported into Maya (version 2014-2016, Autodesk Inc., San Rafael, CA, USA) for animation.

To measure the orientation of the anatomical costovertebral axis formed from the parapophysis and diapophysis, 3D models of the rib-bearing section of the vertebral column were registered in Avizo world space using the Align Principle Axes tool, so that the global *y*-axis was pointed along the vertebral column, and the global *z*-axis pointed dorsally (Fig. 1). Landmarks were placed on the 3D bone models at the locations of the parapophysis and diapophysis along successive thoracic vertebrae in Avizo. Landmark coordinates were exported from Avizo, converted to CSV files, and the *x*, *y* and *z* components of the anatomical axis (*A_x_*_,*y*,*z*_) were calculated, using the following formula:
(1)
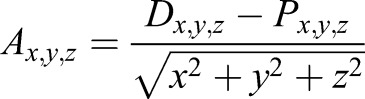


where *D_x_*_,*y*,*z*_ are the *x*, *y* and *z* coordinates of the diapophysis, and *P_x_*_,*y*,*z*_ are the *x*, *y* and *z* coordinates of the parapophysis and so *D_x_*_,*y*,*z*_−*P_x_*_,*y*,*z*_ is the vector between the diapophysis and parapophysis (Fig. 1). This vector was then normalised by dividing by its magnitude 
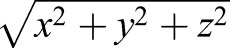
 to calculate the relative *x*, *y* and *z* components of the anatomical axis. These components also indicate the expected relative contributions of pump handle (*x*), calliper (*y*) and bucket handle motion (*z*).

### Marker tracking and XROMM animation

X-ray videos were analyzed using the custom-designed program XMALab (Knörlein et al., 2016) following the standard methodology (software and instructions for use are available at https://bitbucket.org/xromm/xmalab/wiki/Home). Distortion introduced by the X-ray image intensifiers was corrected using standard grid images. The 3D space was calibrated using images of a calibration object with known geometry (a cube with 64 radio-opaque markers). All calibration files are available from the X-ray Motion Analysis Portal (xmaportal.org). XMALab was used for creating rigid-body transformations from the tracked marker positions for marker-based XROMM. Markers were tracked automatically in XMALab, with some manual refinement or interpolation across frames where necessary if automatic tracking failed. The precision of marker tracking, based on the standard deviation of intermarker distances in co-osseous marker pairs (Brainerd et al., 2010), was 0.12±0.01 mm (mean±s.e.m. for 156 marker pairs over 14 trials).

Intermarker distances were measured in XMALab both between co-osseous markers to determine rib segment bending, and between markers on different rib segments to determine whether there was relative motion across the intracostal joints. Intermarker distances were filtered using a low-pass Butterworth filter with a cut-off frequency of 1–2 Hz. Constant intermarker distances indicate that two markers can be modelled as part of the same rigid body. Fluctuations in intermarker distance indicate either bending, if the markers in question are co-osseous, or relative motion of two bones (which should then be modelled as separate rigid bodies), if the markers are on different bones. For comparisons of intermarker distance, a pair of markers implanted in the sternum – which would be assumed *a priori* to display no relative motion – acted as a control. These data determined (1) whether to model the vertebral and intermediate ribs as a single rigid body, or to model all rib segments as separate rigid bodies, and (2) whether to model the cartilaginous intermediate and sternal ribs as flexible or rigid (Brainerd et al., 2016). The vertebral column was divided into marked sections of two or three vertebrae, each of which was animated as a separate rigid body; this was to account for vertebral flexion, which is known to occur during ventilation in *A. mississippiensis* (Claessens, 2009b).

Once all markers had been tracked, rigid-body transformations of the vertebrae, ribs and sternum were calculated and then filtered with a low-pass Butterworth filter with a cut-off frequency of 1–2 Hz. This cut-off is appropriate because the duration of each full breathing cycle was approximately 10 s, so 1–2 Hz is five to 10 times greater than the frequency of motion. Animations were produced by applying the rigid-body transformations for a given marker set to the 3D bone models in Maya. Five full breathing cycles from one inhalation to the next were analysed for all individuals, with kinematic data for various ribs obtained from marker-based analyses. Scientific rotoscoping (Gatesy et al., 2010) – manual positioning of the bones to align with their positions on the X-ray video – did not yield repeatable results because of the lack of external morphology or relative rib segment curvature to guide the alignment process. Compared with *Iguana iguana* (Brainerd et al., 2016), the dorsal ribs of *A. mississippiensis* consist of more segments, each of which more closely approximates a straight cylinder, making it especially difficult to accurately measure long-axis rotation through rotoscoping.

### Joint coordinate systems

**Fig. 2.**
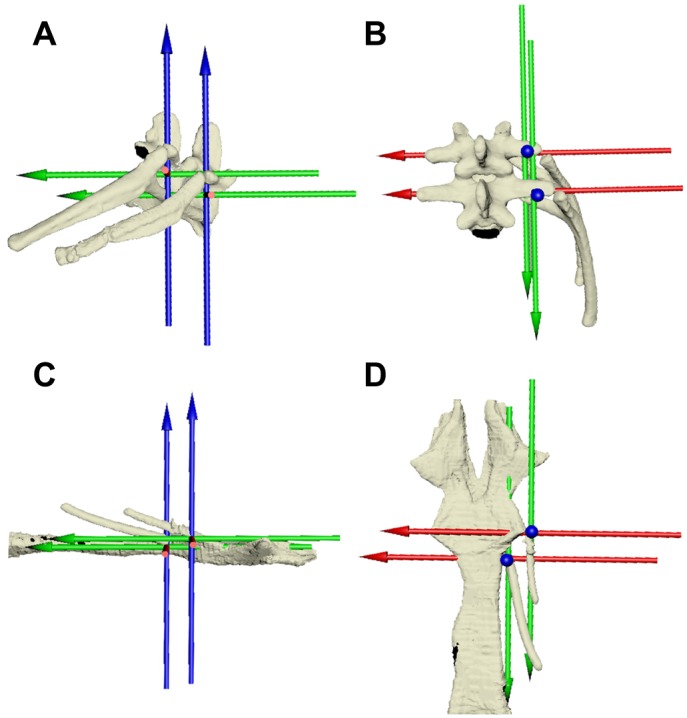
**Joint coordinate systems for the costovertebral and sternocostal joints in *A. mississippiensis*.** (A,B) Costovertebral joint in lateral (A) and dorsal (B) views. (C,D) Sternocostal joint in lateral (C) and dorsal (D) views. Both sets of axes are oriented such that rotation about the *Z*-axis (blue) is bucket handle motion, the *Y*-axis (green) is calliper motion and the *X*-axis (red) is pump handle motion.

Bone kinematic data were extracted from the 3D animations created in Maya using local joint coordinate systems (JCSs). These were established for each joint per rib (Table 1) to measure translation and rotation at that joint. Rotations at JCSs are described in terms of intrinsic Euler angles, with the rotation order set as *ZYX*. The JCSs were oriented relative to the body planes, in order to decompose rotations about the joints into bucket handle, pump handle and calliper motion (Brainerd et al., 2016). The costovertebral and sternocostal JCSs were positioned with the *Z*-axis oriented dorso-ventrally (dorsal is positive), the *Y*-axis oriented cranio-caudally (caudal is positive) and the *X*-axis oriented medio-laterally (left is positive) (Fig. 2). In this position, *Z*-rotation represents bucket handle motion, *Y*-rotation is calliper motion and *X*-rotation is pump handle motion (Fig. 2).

**Table 1.**
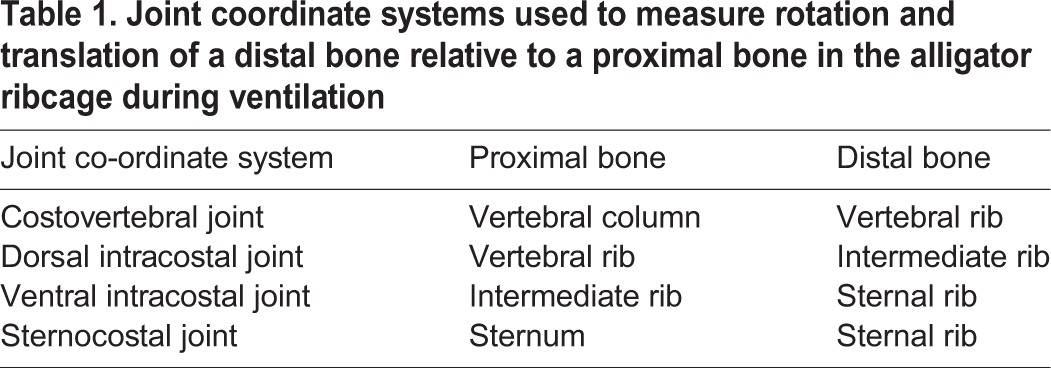
**Joint coordinate systems used to measure rotation and translation of a distal bone relative to a proximal bone in the alligator ribcage during ventilation**

**Fig. 3.**
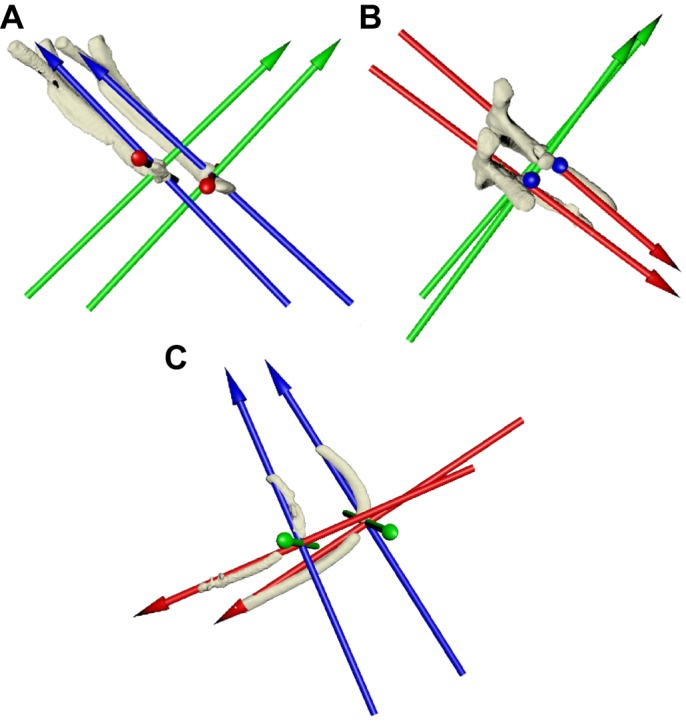
**Joint coordinate systems for the dorsal and ventral intracostal joints in *A. mississippiensis*.** (A,B) Dorsal intracostal joint in lateral (A) and dorsal (B) views. (C) Ventral intracostal joint in oblique view. Axes are oriented such that the *X*-axis (red) is long-axis rotation of the distal bone, the *Z*-axis (blue) lies in the plane of the proximal bone and the *Y*-axis (green) is orthogonal to these two.

For the dorsal and ventral intracostal joints, the JCS was oriented so that the *X*-axis was aligned along the length of the distal rib (intermediate and sternal, respectively), and the *Z*-axis lay in the plane of the proximal rib (vertebral and intermediate, respectively) at maximum inhalation, with the *Y*-axis orthogonal to both (Fig. 3). In this case, the *X*-axis measures long-axis rotation of the distal rib, the *Z*-axis measured abduction–adduction of the distal rib relative to the proximal rib, and the *Y*-axis measured depression–elevation of the distal rib relative to the proximal rib (Brainerd et al., 2016).

For all these local co-ordinate systems, the positions of the bones at maximum inhalation were chosen as the zero pose (i.e. starting position) for each joint, rather than using a non-anatomical reference pose as in previous XROMM studies (Baier et al., 2013; Brainerd et al., 2016). As breaths varied in duration and magnitude, in order to better compare breaths of differing lengths, a standard resampling procedure was applied (Gidmark et al., 2014; Brainerd et al., 2016). Data were imported into R (http://www.r-project.org) and resampled to 100 points, each point being 1% of the breathing cycle, and zeroed to the mean angle of each rotational degree of freedom.

### Axis-angle decomposition

The orientation of the anatomical costovertebral joint axes obtained from morphological measurements provides a prediction for how the vertebral ribs will rotate in terms of the proportions of pump, bucket and calliper motion. However, the joint axis vectors are not directly comparable with the Euler angles obtained from JCSs. Therefore, to test whether morphology really can predict motion, the rotation of the ribs about the costovertebral joint was converted from the Euler angle representation to an axis-angle representation. Here, rotation of the ribs relative to the vertebral column is represented as a rotation (θ) about a single vector with *x*, *y* and *z* components, which are directly comparable to the *x*, *y* and *z* components of the anatomical joint axes.

Frames representing peak inhalation and exhalation were selected from the splined-averaged data and the difference was calculated, to give the ‘total’ rotation of the ribs across the breathing cycle, analogous to some human studies (e.g. Beyer et al., 2014). The Euler angles for rib rotation about the costovertebral joint were then converted to an axis-angle representation using the spincalc function (J. Fuller) in MATLAB (version 2016; MathWorks, Natick, MA, USA). The orientations of both vectors – the predicted anatomical axis of rotation and the *in vivo* axis measured from the XROMM data – are given as angles between the vector and the three anatomical body planes (sagittal, coronal and transverse for the *x*, *y* and *z* components, respectively).

If the hinge model of rib motion is correct, we would expect that the *in vivo* rotation axis of the rib would have the same orientation as that which runs through the parapophysis and diapophysis. Linear regressions of the expected angle (based on bony morphology) and measured angle (from the XROMM data) between each body plane and the axis of rotation were performed in R.

## RESULTS

### Joint mobility and rigid bodies

**Fig. 4.**
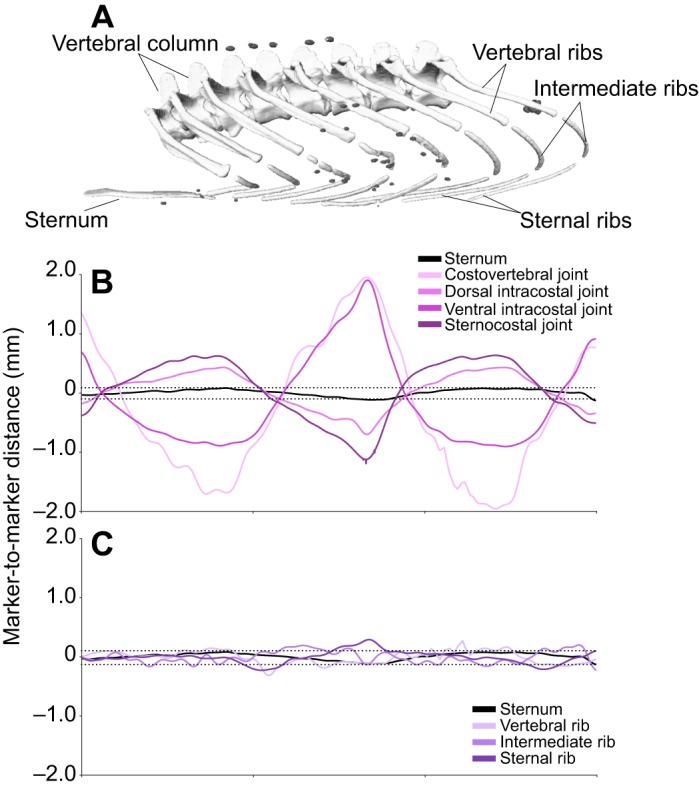
**Relative motions of radio-opaque markers in the vertebral, intermediate and sternal ribs over the course of two breaths in *A. mississippiensis*.** (A) Mesh model of specimen Ag5L5, showing bones and markers. (B) Changes in filtered inter-marker distances across joints (between markers on different bones). (C) Changes in filtered inter-marker distances between co-osseous markers. Dotted lines show marker tracking precision (±0.12 mm). For the vertebral, intermediate and sternal ribs, the changes between co-osseous markers are low, suggesting no significant rib bending. Across both the dorsal and the ventral intra-costal joints, the changes in intermarker distance are relatively high, indicating significant motion across joints, and so each rib segment was treated as a separate rigid body.

Among all three rib segments there were relatively large inter-marker distance changes, and the pattern of these fluctuations corresponded to the breathing pattern of the animal (Fig. 4B). This indicates flexion at the intracostal joint(s), consistent with previous work (Claessens, 2009b), and therefore each individual rib segment was modelled as a separate rigid body. Both the ossified vertebral ribs and the cartilaginous sternal and intermediate ribs in *A. mississippiensis* showed no evidence of noticeable bending. There were slight fluctuations in the intermarker distance between co-osseous markers consistent with the breathing pattern, suggestive of bending during ventilation; however, this effect was only minor, and mostly fell within the range of marker tracking precision (Fig. 4C), so all rib segments – both the ossified and cartilaginous ones – were treated as rigid bodies here.

### The costovertebral joint

**Fig. 5.**
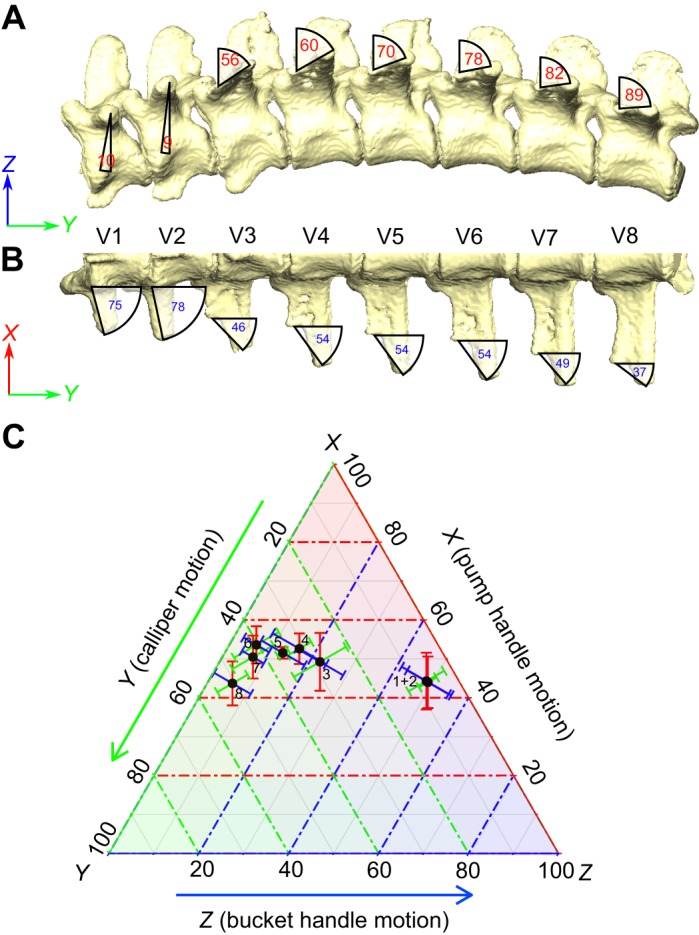
**Axis-angle decomposition of an axis connecting the diapophysis and parapophysis for costovertebral joints 1–8 in *A. mississippiensis*.** (A,B) Thoracic vertebral column in lateral (A) and ventral (B) views showing the projected angles between the diapophysis and parapophysis in the sagittal and transverse planes. (C) Ternary plot showing mean orientation of each costovertebral joint in *A. mississippiensis* expressed as relative percentages of *X*, *Y* and *Z* (note the *X*-axis begins at 20%). Error bars show ±s.d. (*n*=4 individuals). The orientations of the joints predict the relative amounts of pump handle, calliper and bucket handle motion expected.

Changes in the orientation of the costovertebral joints in *A. mississippiensis*, expressed as the relative *X*, *Y* and *Z* components of each (averaged across multiple individuals) are shown in Fig. 5. The first two costovertebral joints have high *X* and *Z* components, and so pump and bucket handle motion are predicted to dominate (Fig. 5). From the third costovertebral joint, where the parapophysis shifts onto the transverse process, the *X* component stays relatively constant, but the *Z* component decreases and the *Y* component increases. Hence, increasing calliper motion at the expense of bucket handle motion is predicted in ribs 3–8 (Fig. 5C).

**Fig. 6.**
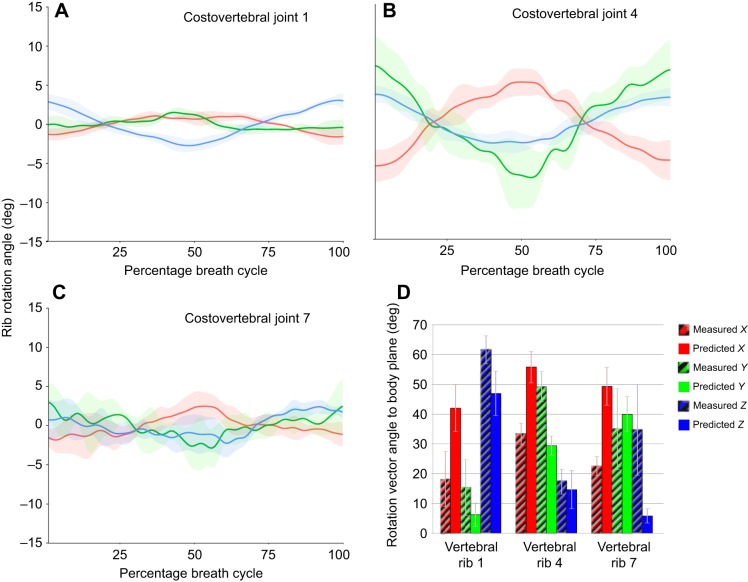
***In vivo* motions at the costovertebral joints in *A. mississippiensis* from XROMM animations.** Pump handle (red), calliper (green) and bucket handle (blue) rotation of vertebral ribs relative to the vertebrae with joint coordinate system orientation from Fig. 2A,B. (A) Costovertebral joint 1. (B) Costovertebral joint 4. (C) Costovertebral joint 7. Graphs show means±s.d. for five breaths. (D) Barplot showing angles between body planes and vectors of rotation obtained through axis-angle decomposition for vertebral ribs 1, 4 and 7, comparing the angles measured *in vivo* with those predicted based on joint anatomy. Error bars show ±s.d.

From XROMM animations, the *in vivo* motion of the first vertebral rib about the costovertebral joint was dominated by bucket handle motion, with minor contributions from pump and calliper motion (Fig. 6A, Table S2). During exhalation, the ribs rotated caudo-medially and slightly caudo-dorsally (Movies 2 and 3), folding inwards and decreasing thoracic volume (decreasing bucket handle and increasing pump handle, with axis polarity set using the right-hand rule; see JCS in Fig. 2A,B). During inhalation, the ribs rotated out again cranio-ventrally and cranio-laterally, expanding the ribcage (Movies 2 and 3). Moving caudally in the ribcage to the fourth rib, once the parapophysis has shifted onto the transverse process, rotation of the ribs is mostly made up of calliper and pump handle motion, but there is still a clear bucket handle component (Fig. 6B, Table S2). Additionally, the actual range of motion is higher in the fourth vertebral rib as opposed to the first (Fig. 6B). In the caudal-most ribs, however, bucket handle, calliper and pump handle motions all occur in approximately equal measure, and the actual range of motion is lower than in the middle part of the ribcage (comparing rib 7 with rib 4) (Fig. 6C, Table S2).

Following the axis-angle decomposition, comparing the axis orientations measured *in vivo* with those predicted from joint anatomy, it appears that whilst the general trends match – decreasing importance of bucket handle motion and an increase in calliper motion – the precise details differ substantially (Fig. 6D). Predicted axes of rotation from joint anatomy overestimated the *X* component of the vector describing the axis, and underestimated the *Z* component; in terms of Euler angles, these represent an overestimate of pump handle motion and an underestimate of bucket handle motion. Additionally, the discrepancies seemed to be most pronounced in the caudal ribs (Fig. 6D, rib 7), where the *in vivo* axis of rotation has almost equal *x*, *y* and *z* components.

**Fig. 7.**
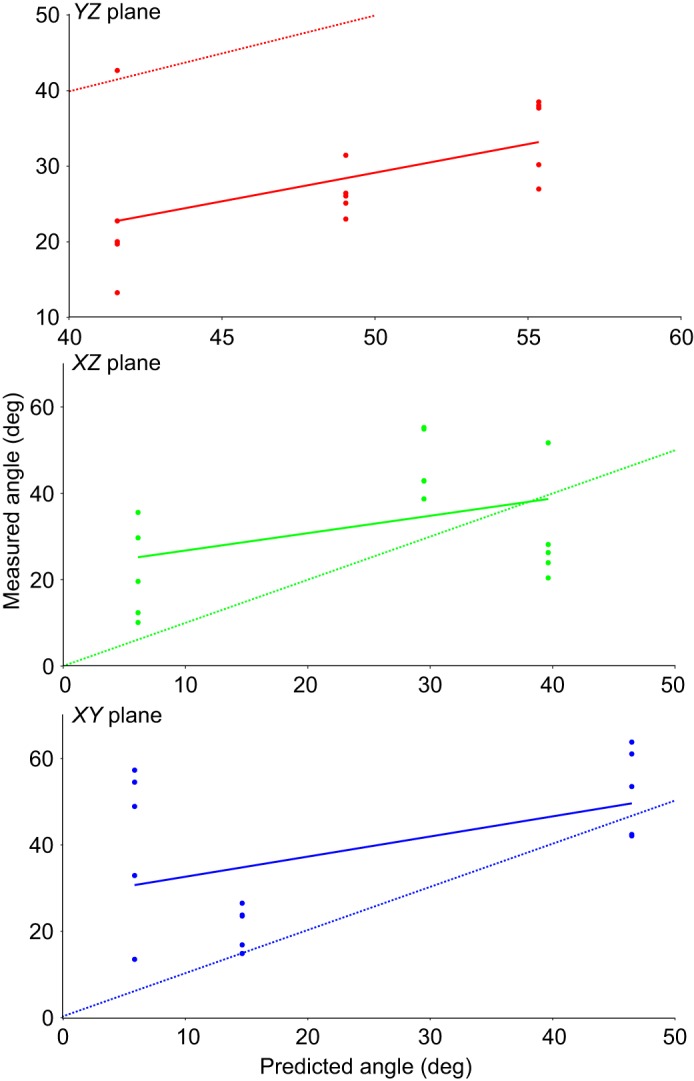
**Relationship between predicted and measured motions at the costovertebral joints in *A. mississippiensis*.** Scatterplots with regression lines showing the relationship between the orientation of the vertebral rib axis of rotation as predicted from joint morphology and that observed *in vivo* using XROMM. Orientation of each axis is measured as an angle from the (A) *YZ* (sagittal), (B) *XZ* (coronal) and (C) *XY* (transverse) planes for costovertebral joints 1, 4 and 7. The dashed lines show lines of identity (predicted angle=measured angle).

Even though there appears to be the absence of a one-to-one relationship between the predictions of bony morphology and the actual rotations of the ribs observed via XROMM, this does not mean the two are not related. Linear regression results show significant relationships between predicted and measured values for all three axes (*X*: *P*=0.004; *Y*: *P*=0.017; *Z*: *P*=0.002), although the relationship is stronger for pump (*R*^2^=0.4494; Fig. 7A) and bucket handle motion (*R*^2^=0.4925; Fig. 7C) than for calliper motion (*R*^2^=0.3139; Fig. 7B). Additionally, the regression results show that morphology alone does significantly underestimate bucket handle motion (intercept greater than zero, *P*=0.011; Fig. 7C). However, the overestimate of pump handle motion was only suggestive, not statistically significant (intercept less than zero, *P*=0.079; Fig. 7A).

### The intracostal and sternocostal joints

**Fig. 8.**
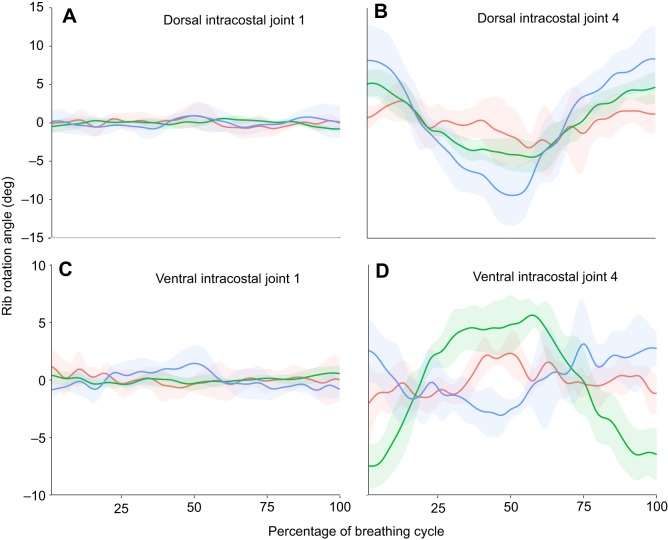
***In vivo* motions at the intracostal joints in *A. mississippiensis* from XROMM animations.** Long-axis rotation (red), depression–elevation (green) and abduction–adduction (blue) rotation of the dorsal intracostal joint between the vertebral rib and intermediate rib (joint coordinate system in Fig. 3A,B) and the ventral intracostal joint between the intermediate rib and the sternal rib (joint coordinate system in Fig. 3C). (A) Dorsal intracostal joint 1. (B) Dorsal intracostal joint 4. (C) Ventral intracostal joint 1. (D) Ventral intracostal joint 4. Graphs show means±s.d. (*N*=5 breaths).

The dorsal intracostal joint is mobile in *A. mississippiensis*, although this mobility is most pronounced in the more caudal ribs (Fig. 8A, Table S3). Whilst there is very little measurable rotation about this joint in the first dorsal rib, more caudally there is definite abduction–adduction (*Z*-axis rotation) and depression–elevation (*Y*-axis rotation) of the intermediate rib relative to the vertebral rib (Fig. 8B, Table S3). Long-axis rotation of the intermediate rib relative to the vertebral rib is low in both the cranial and caudal ribs (Fig. 8A,B). The ventral intracostal joint shows a similar pattern, with little motion in the cranial ribs, but becoming much more pronounced in the caudal ribs (Fig. 8C,D, Table S4). Some long-axis (*X*-axis) rotation of the sternal rib relative to the intermediate rib does occur at the ventral intracostal joint (Fig. 8C,D, Table S4); however, this is still a relatively minor component of the motion, which is dominated by abduction–adduction (*Z*-axis) and depression–elevation (*Y*-axis).

**Fig. 9.**
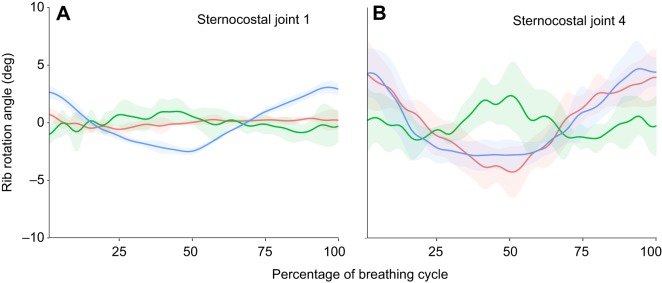
***In vivo* motions at the sternocostal joints in *A. mississippiensis* from XROMM animations.** Pump handle (red), calliper (green), and bucket handle (blue) rotation about the sternocostal joint (joint coordinate system in Fig. 2C,D) in (A) sternal rib 1 and (B) sternal rib 4. Graphs show means±s.d. (*N*=5 breaths).

At the first sternocostal joint, bucket handle motion dominates, with very little pump or calliper motion (Fig. 9A, Table S5). However, moving more caudally in the ribcage, bucket handle motion remains prominent, but there is also considerable pump and calliper motion as well (Fig. 9B, Table S5). Additionally, similar to the intracostal joints, the ribs in the caudal part of the ribcage show greater variability in how they rotate (larger confidence intervals on each frame) (Figs 8 and 9).

## DISCUSSION

### Does costovertebral joint morphology predict vertebral rib motion?

The transition of the parapophysis from the vertebral centrum to the transverse process has been well documented within crocodylians (Claessens, 2009b; Schachner et al., 2009), and also in fossil archosaurs (Ewer, 1965; Piechowski and Dzik, 2010; Schachner et al., 2011; Geist et al., 2014). This bicondylar articulation is thought to act as a hinge joint (Claessens, 2009b), and so the cranial vertebral ribs would be expected to move mostly with pump and bucket handle motion, and the caudal ribs mostly with pump and calliper motion, although until now the explicit consequences of this shift for rib rotation have not been experimentally tested.

Whilst the morphology of the costovertebral joints did predict overall patterns of rib motion (Fig. 7), there were still substantial differences between these predictions and rib kinematics observed *in vivo*, notably that the anatomical axes seemed to overestimate the degree of pump handle motion and underestimate bucket handle motion across the ribcage (Fig. 6D). In the cranial ribs, this may be due to increased flexion of the vertebral column *in vivo* inclining the first two vertebrae cranially and so tilting the axis of rotation towards the vertical. Vertebral flexion does form part of the breathing mechanism in crocodylians, albeit a small one (Claessens, 2009b), and few other animals have an entirely horizontal backbone; therefore, when attempting to predict rib motion based on costovertebral joint morphology, the orientation and motion of the vertebrae themselves must also be considered.

In the more caudal ribs, *in vivo* rib rotation was apparently unconstrained by the hinge-axis of the parapophysis and diapophysis and generally was more equal about all three axes than might be expected (Fig. 6). The hypothesis that bicapitate ribs in animals such as crocodylians are constrained to a single hinge-like axis of rotation assumes that there is no translation at these articulations, and therefore the joint centre of rotation lies between the parapophysis and the diapophysis. The morphology of the parapophyseal facets in the third thoracic vertebra onwards – flatter and more medio-laterally inclined – has been suggested to permit some translation of the rib capitulum (Claessens, 2009b), and previous descriptions have the more caudal thoracic ribs articulating with the vertebral column ‘principally by their tubercula’ (Hoffstetter and Gasc, 1969). Given these observations, the assumptions of the ‘hinge’ model for rib motion in crocodylians (and possible fossil archosaurs) may not be valid even on a simple osteological basis, and the axis-angle decomposition presented here shows that the ‘true’ axis of rib rotation does not necessarily align closely with that predicted by joint morphology.

Further complicating factors include the presence of soft tissues both at the costovertebral joint and between adjacent ribs. Following the migration of the parapophysis onto the transverse process, the soft tissue attachment between the vertebral ribs and vertebral column is weakened (Frey, 1988), and so might be more permissive of motions outside those predicted by the ‘anatomical axis’ of the diapophysis and parapophysis. By contrast, the two most cranial thoracic ribs are strongly attached to the vertebral column (Frey, 1988). It could also be argued that given that successive ribs are linked together by the intercostal musculature (Gans and Clark, 1976; Frey, 1988), the motion of one rib will affect the motion of the ribs to either side of it, resulting in greater uniformity of rib motion across the ribcage – which is what is observed *in vivo* – compared with what might be predicted.

### Do the intermediate ribs move and how much?

The results of the intermarker distance measures (Fig. 4) show that the intermediate ribs do move relative to the vertebral ribs. There is some mobility at the dorsal and ventral intracostal joints, particularly in the more caudal ribs (Fig. 8). Both of these joints are monocondylar, and so theoretically unconstrained in how they can rotate. Preliminary XROMM analysis of the first two dorsal ribs suggested little or no motion at the dorsal intracostal joint during breathing (Moritz and Brainerd, 2013), but the more complete analysis here shows that the ribs are functionally tripartite, with the greatest dorsal intracostal joint mobility in the caudal ribs.

The presence of a mobile dorsal intracostal joint also contrasts with other sauropsids; for example, the tegu *Salvator merianae* has an immobile dorsal intracostal joint, with the vertebral and intermediate ribs functioning as a single unit (Capano et al., 2016). In *I. iguana*, which has bipartite ribs, there is no motion at the joint between the osseous and cartilaginous parts of the rib (which may be considered analogous to the dorsal intracostal joint in *S. merianae* and *A. mississippiensis*). Comparing lepidosaurs with archosaurs, there are some differences in the morphology of the distal vertebral ribs, which may be linked to differences in mobility at the (dorsal) intracostal joint. In lepidosaurs – including *I. iguana* (Brainerd et al., 2016), *S. merianae* (Capano et al., 2016) and *Sphenodon* (R.J.B., unpublished observations) – the distal tips of the vertebral ribs have a concave surface, and into this concavity fit cartilaginous plugs (e.g. *I. iguana*) or bony intermediate ribs (e.g. *S. merianae*, *Sphenodon*). No such concavity exists in crocodylians, which may be related to the presence of a mobile dorsal intracostal joint in these taxa.

### Motion of the sternal ribs

The rotations occurring at the sternocostal joint differ substantially between the cranial and caudal ribs. The more complex motions of the more caudal sternal ribs are presumably related both to the changes in the motion of the vertebral ribs, and the increased mobility of both the dorsal and ventral intracostal joints. The sternocostal joint is monocondylar in crocodylians, where the cartilaginous rib tip articulates with the cartilage of the sternum (Claessens, 2015); this joint type is theoretically unconstrained and should be capable of motion using all three rotational degrees of freedom (Brainerd et al., 2016), making it difficult to predict *a priori* how the sternal ribs will move based on the anatomy of the sternocostal joints.

In the first dorsal rib of *A. mississippiensis*, there is very little motion at either the dorsal or ventral intracostal joint (Fig. 8A,C), and so the vertebral, intermediate and sternal ribs could be said to act as almost a single unit. Because the first costovertebral joint is dominated by bucket handle motion, then we might expect the first sternocostal joint to be dominated by bucket handle motion, as can be seen in Fig. 9A. A similar situation is found in the lepidosaur *I. iguana*; despite having the potential to rotate about all three main axes, motions at the sternocostal joint are dominated by bucket handle motion, as are motions at the costovertebral joint (Brainerd et al., 2016). In the more caudal ribs of *A. mississippiensis*, the vertebral ribs move in a more complex fashion, and so this would impact the motion of the sternal ribs. Added to this, the dorsal and ventral intracostal joints are much more mobile in the more caudal ribs, and so the additional degrees of freedom present in the ribcage as a result of having mobile intermediate ribs become apparent; motion of the sternal rib is no longer dependent simply on how the vertebral rib moves.

For breathing in amniotes, the ventral ribcage seems to play an important role. In crocodylians, in addition to the results presented here, Gans and Clark (1976) reported significant activity in the parasternal intercostal muscles during inhalation. In birds, the motion of the sternum and sternal ribs plays a crucial role in ventilating the lung–air sac system (Codd et al., 2005; Tickle et al., 2007; Claessens, 2009a), and in stem birds and non-avian dinosaurs, the ‘cuirassial basket’ – composed of the sternum and gastralia – potentially played a role in ventilation (Carrier and Farmer, 2000; Claessens, 2004a; Codd et al., 2008). In other diapsids, preliminary analysis of *I. iguana* suggests that the parasternal intercostals are important drivers of thoracic expansion (Brainerd, 2015). In mammals, the parasternal intercostals have the greatest mechanical advantage for inspiration (De Troyer et al., 2005), and they retain a respiratory motor pattern during locomotion, when the other intercostals switch to a locomotor activity pattern (Deban and Carrier, 2002).

### Costal aspiration in fossil archosaurs

The results of this XROMM analysis have implications for how we reconstruct rib motion and breathing mechanics in fossil archosaurs. Modern crocodylians retain several aspects of the axial skeleton which are considered ‘primitive’, e.g. the shift in the position of the parapophysis (Ewer, 1965; Schachner et al., 2011), and so are assumed to represent a good model for the ancestral archosaur (Claessens, 2015). The costovertebral joint is a main osteological correlate of rib mobility (Janis and Keller, 2001; Schachner et al., 2009; Brainerd, 2015; Claessens, 2015) and so a proper understanding of the relationship between rib and vertebral shape and ribcage mobility is key to accurately reconstructing the mechanics of ventilation in extinct archosaurs. Although the results presented here suggest that joint morphology alone cannot accurately predict vertebral rib motion precisely, they illuminate where the errors may lie (e.g. underestimating bucket handle motion), and so these errors can be corrected for when attempting to accurately reconstruct fossils. Other aspects of ribcage anatomy (e.g. ligamental scars; Hirasawa, 2009) can also be used to further constrain estimates of rib motion and thoracic volume change in extinct taxa.

The presence of mobile intermediate ribs in extant crocodylians raises the possibility that they were also present in fossil archosaurs. Additionally, the role played by the sternal ribs emphasises the importance of the ventral trunk during ventilation. Currently, however, these questions cannot be investigated directly, as the intermediate ribs, sternal ribs and sterna of most extinct taxa were cartilaginous and so have failed to fossilise. Even in exceptional preservation settings, cartilage is less frequently preserved than other soft tissues (Zhou et al., 2003; O'Connor et al., 2014), and although a few examples of cartilage preservation are known (O'Connor et al., 2014), no specimens preserve a whole ribcage with cartilaginous elements. Complete ribcages are known from fossil maniraptoran theropods and pterosaurs, both of which preserve ossified bipartite ribs with bony vertebral and sternal segments (Codd et al., 2008; Claessens et al., 2009), meaning that the intermediate ribs were absent in these groups, and this likely represents the transition from a tripartite to a bipartite ribcage (Claessens, 2015).

Despite the lack of fossils, the presence of a mobile, tripartite ribcage in more basal archosaurs may still be inferred based on developmental data from extant taxa and phylogenetic bracketing (Witmer, 1995). Crocodylians have tripartite ribs and the bipartite ribs of birds are derived from three parent tissues (Aoyama et al., 2005) and ossify from three centres (one for the sternal and two for the vertebral rib; Maxwell, 2008). Therefore, it may be assumed that the ancestral archosaur did possess a tripartite ribcage, and so at least some fossil groups would have possessed (potentially mobile) intermediate ribs (Claessens, 2015). Analysis of the morphology of the distal vertebral ribs may provide information on the morphology and mobility of the intermediate and sternal ribs (Janis and Keller, 2001; Brainerd, 2015). Although the ventral ribcage is not preserved in most fossils, in life it would have been connected to the vertebral ribs; even if motion of the sternal ribs is a major driver of ventilation, the vertebral ribs and costovertebral joints still contain information about rib motion (Brainerd, 2015).

### Concluding remarks

Understanding the form–function relationships between ribcage morphology (including the costal joints) and the motion of the ribs and sternum is crucial for our understanding of ventilation in all amniote groups. Costal aspiration is the primitive breathing mode for Archosauria and so forms the functional context in which subsequent innovations in breathing mechanics – the crocodylian hepatic piston or the evolution of the highly modified avian ribcage – evolved. Crocodylians, despite having their own derived specialisations, still represent our best extant model for the early archosaur ribcage and so understanding the mechanics of costal aspiration in these taxa will aid our understanding of the evolution of ventilation and the respiratory system in both the crocodylian and avian lineages. The results presented here seek to bridge the gap between predictions made from bony morphology and actual motions of ribs *in vivo*. This not only provides information on the ventilation mechanics of extant crocodylians, but also serves as a vital ‘reality check’, ground-truthing results from, and testing the assumptions of, models of breathing mechanics in fossil archosaurs.
